# Impact of Systemic Treatments on Outcomes and Quality of Life in Patients with RAS-Positive Stage IV Colorectal Cancer: A Systematic Review

**DOI:** 10.3390/diseases12040079

**Published:** 2024-04-20

**Authors:** Vlad Braicu, Pantea Stelian, Lazar Fulger, Gabriel Verdes, Dan Brebu, Ciprian Duta, Camelia Fizedean, Flavia Ignuta, Alexandra Ioana Danila, Gabriel Veniamin Cozma

**Affiliations:** 1 Doctoral School, “Victor Babes” University of Medicine and Pharmacy Timisoara, Eftimie Murgu Square 2, 300041 Timisoara, Romania; braicu.vlad@umft.ro (V.B.); flavia.ignuta@umft.ro (F.I.); alexandra.danila@umft.ro (A.I.D.); 2 Department of General Surgery, “Victor Babes” University of Medicine and Pharmacy Timisoara, Eftimie Murgu Square 2, 300041 Timisoara, Romania; pantea.stelian@umft.ro (P.S.); lazar.fulger@umft.ro (L.F.); verdes.gabriel@umft.ro (G.V.); brebu.dan@umft.ro (D.B.); duta.ciprian@umft.ro (C.D.); 3 Methodological and Infectious Diseases Research Center, “Victor Babes” University of Medicine and Pharmacy Timisoara, Eftimie Murgu Square 2, 300041 Timisoara, Romania; 4 Department of Anatomy and Embryology, Discipline of Pulmonology, “Victor Babes” University of Medicine and Pharmacy Timisoara, Eftimie Murgu Square 2, 300041 Timisoara, Romania; 5 Discipline of Surgical Semiology I and Thoracic Surgery, Department of Surgery I, “Victor Babes” University of Medicine and Pharmacy Timisoara, Eftimie Murgu Square 2, 300041 Timisoara, Romania; gabriel.cozma@umft.ro; 6 Thoracic Surgery Research Center, “Victor Babes” University of Medicine and Pharmacy Timisoara, Eftimie Murgu Square 2, 300041 Timisoara, Romania

**Keywords:** quality of life, oncology, colorectal cancer, metastatic disease

## Abstract

This systematic review critically evaluates the impact of systemic treatments on outcomes and quality of life (QoL) in patients with RAS-positive stage IV colorectal cancer, with studies published up to December 2023 across PubMed, Scopus, and Web of Science. From an initial pool of 1345 articles, 11 relevant studies were selected for inclusion, encompassing a diverse range of systemic treatments, including panitumumab combined with FOLFOX4 and FOLFIRI, irinotecan paired with panitumumab, regorafenib followed by cetuximab ± irinotecan and vice versa, and panitumumab as a maintenance therapy post-induction. Patient demographics predominantly included middle-aged to elderly individuals, with a slight male predominance. Racial composition, where reported, showed a majority of Caucasian participants, highlighting the need for broader demographic inclusivity in future research. Key findings revealed that the addition of panitumumab to chemotherapy (FOLFOX4 or FOLFIRI) did not significantly compromise QoL while notably improving disease-free survival, with baseline EQ-5D HSI mean scores ranging from 0.76 to 0.78 and VAS mean scores from 70.1 to 74.1. Improvements in FACT-C scores and EQ-5D Index scores particularly favored panitumumab plus best supportive care in KRAS wild-type mCRC, with early dropout rates of 38–42% for panitumumab + BSC. Notably, cetuximab + FOLFIRI was associated with a median survival of 25.7 months versus 16.4 months for FOLFIRI alone, emphasizing the potential benefits of integrating targeted therapies with chemotherapy. In conclusion, the review underscores the significant impact of systemic treatments, particularly targeted therapies and their combinations with chemotherapy, on survival outcomes and QoL in patients with RAS-positive stage IV colorectal cancer, and the need for personalized treatment.

## 1. Introduction

Colorectal cancer (CRC) stands as the third most commonly diagnosed malignancy and the second leading cause of cancer-related mortality worldwide [1]. The prognosis for stage IV colorectal cancer, characterized by distant metastases, remains poor, with a five-year survival rate of approximately 14% [2]. Among the molecular aberrations driving CRC progression, mutations in the RAS oncogene family, including KRAS and NRAS, are of significant clinical importance [3,4]. These mutations are detected in up to 50% of colorectal cancer cases, and are pivotal in the tumor’s behavior, response to therapy, and overall patient prognosis [5].

The introduction of systemic treatments, encompassing chemotherapy, targeted therapy, and immunotherapy, has transformed the therapeutic landscape for advanced-stage cancers [6,7,8,9,10,11,12]. Particularly, the efficacy of anti-epidermal growth factor receptor (EGFR) therapies, such as cetuximab and panitumumab, is markedly influenced by the RAS mutation status in stage IV CRC [13]. Patients harboring RAS mutations exhibit resistance to some therapeutic agents, underscoring the necessity for a tailored treatment approach based on genetic profiling [14]. Consequently, the identification of RAS mutations has become a cornerstone in the decision-making process for selecting appropriate systemic treatments [13,14].

Despite advancements in treatment strategies, the impact of systemic therapies and surgery on the outcomes and quality of life (QoL) of patients with cancer remains an important area of study focus, based on staging, treatment-resistance, among other influencing factors [15,16,17,18,19,20]. Quality of life, an integral component of cancer care, can be measured by various standardized scales that encompass physical, psychological, and social domains affected by the disease and its treatment [21,22]. Given the aggressive nature of stage IV CRC and the challenges associated with managing RAS-mutant tumors, investigating the outcomes and QoL in this patient population is paramount.

Real-world data highlight the complexity of treating RAS-positive CRC, demonstrating that patients with RAS-mutant CRC have a median overall survival ranging from 20 to 30 months when treated with current standard systemic therapies, compared to longer survival in those with RAS wild-type tumors [23]. Furthermore, the adverse effects associated with systemic treatments, including chemotherapy-induced neuropathy and the dermatological toxicities from targeted therapies, can significantly impair patients’ quality of life [24,25].

Therefore, this systematic review aims to critically evaluate the existing literature on the outcomes and quality of life of patients with RAS-positive stage IV colorectal cancer following systemic treatment. By synthesizing data from clinical trials, observational studies, and real-world evidence, this review seeks to provide a comprehensive understanding of the efficacy and impact of systemic therapies in this distinct patient cohort, ultimately guiding future research and clinical practice towards improving both survival and quality of life for patients with advanced RAS-mutant colorectal cancer.

## 2. Materials and Methods

### 2.1. Protocol and Registration

The study was justified by the need to address the specific evolution of the disease and the associated quality of life under systemic treatments for late-stage (metastatic) RAS-positive colorectal cancer, a topic not previously covered by other systematic reviews, emphasizing the different and unique treatment approaches and disease progression in this distinct patient group. To conduct the systematic review, this study employed a detailed search strategy across PubMed, Scopus, and Web of Science. The literature search aims to include publications up to December 2023, thereby capturing the most recent and pertinent studies in this field.

The search strategy incorporated a broad range of keywords and phrases relevant to the objectives of the study, with a focus on systemic treatments and their impact on patients with RAS-positive stage IV colorectal cancer. Key search terms comprised: “RAS-positive”, “KRAS”, “NRAS”, “wild type”, “colorectal cancer”, “metastatic colorectal cancer”, “stage IV colorectal cancer”, “systemic treatment”, “chemotherapy”, “targeted therapy”, “immunotherapy”, “anti-EGFR therapy”, “survival outcomes”, “quality of life”, “adverse effects”, and “treatment efficacy”.

Boolean operators (AND, OR, NOT) were used to effectively combine and refine search terms. The search string included some of the following combinations: ((“RAS-positive” OR “KRAS” OR “NRAS”) AND (“colorectal cancer”) AND (“stage IV”) AND (“systemic treatment” OR “chemotherapy” OR “targeted therapy” OR “immunotherapy” OR “anti-EGFR therapy”) AND (“survival outcomes” OR “quality of life” OR “adverse effects” OR “treatment efficacy”)).

Adhering to the Preferred Reporting Items for Systematic Reviews and Meta-Analyses (PRISMA) guidelines [26], this protocol has been designed to ensure a structured, transparent, and reproducible methodology. To further promote the accessibility and transparency of our research process and findings, the review has been registered with the Open Science Framework register of systematic reviews, providing open access to our methodologies and anticipated outcomes. The registration number for this review is osf.io/jpcxr.

### 2.2. Eligibility Criteria

The inclusion criteria were considered as follows: (1) study population: studies must involve patients diagnosed with stage IV colorectal cancer harboring RAS mutations (including KRAS and NRAS). (2) Focus on systemic treatments and outcomes: The research must specifically examine the impact of systemic treatments (chemotherapy, targeted therapy, immunotherapy) on survival outcomes and quality of life in the specified patient population. This includes studies assessing treatment efficacy, survival rates (overall survival, progression-free survival), quality of life measures, and adverse effects associated with the treatments. (3) Types of studies: The review will incorporate a diverse range of study designs, including randomized controlled trials (RCTs), observational studies, clinical trials, cohort studies, case–control studies, and cross-sectional studies. Qualitative studies offering detailed insights into patient experiences with systemic treatments will also be considered. (4) Outcome measures: studies that employ validated instruments or clearly defined parameters to evaluate survival outcomes, quality of life, and treatment-related adverse effects. And (5) language: only peer-reviewed articles published in English will be included to ensure the comprehensiveness and quality of the review and analysis.

The exclusion criteria comprised the following: (1) Non-human studies: any research not involving human subjects, such as in vitro studies or animal models specifically focusing on colorectal cancer, will be excluded to maintain the focus on human patient experiences and outcomes; (2) broad cancer focus: studies not exclusively examining patients with RAS-positive stage IV colorectal cancer, or those that do not differentiate the effects of systemic treatments on this specific subgroup, will be excluded; (3) lack of specific outcomes: research that does not provide clear, quantifiable outcomes related to survival rates, quality of life, and adverse effects of treatments, or lacks sufficient detail for an in-depth analysis, will be omitted; and (4) grey literature: to uphold the credibility and reliability of the data included in the review, grey literature, including non-peer-reviewed articles, thesis dissertations, conference proceedings, narrative reviews, systematic reviews, meta-analyses, commentaries, and editorials, will be excluded.

### 2.3. Definitions

As defined by the American Joint Committee on Cancer (AJCC) TNM classification, stage IV colorectal cancer indicates that the cancer has metastasized beyond the primary site to distant organs or tissues, such as the liver, lungs, peritoneum, or distant lymph nodes [27]. Moreover, quality of life (QoL) in patients with colorectal cancer can be assessed using various validated instruments designed to measure the physical, psychological, and social aspects affected by the disease and its treatment. These include: The European Organization for Research and Treatment of Cancer Quality of Life Questionnaire (EORTC QLQ-C30), its colorectal cancer-specific module (EORTC QLQ-CR29), and The Functional Assessment of Cancer Therapy-Colorectal (FACT-C) [28].

RAS mutations refer to alterations in any of the genes in the RAS family (KRAS, NRAS, HRAS) that lead to the production of a constitutively active RAS protein, driving oncogenic signaling pathways that promote cell growth, differentiation, and survival [29]. In colorectal cancer, mutations in KRAS and NRAS are of particular clinical significance, as they are associated with resistance to anti-EGFR therapies. These mutations are somatic, meaning they occur in cells of the body other than sperm or egg cells, and are not inherited but acquired during one’s lifetime. The presence of RAS mutations is a critical determinant in the selection of targeted therapies for colorectal cancer, influencing treatment efficacy and patient outcomes.

### 2.4. Data Collection Process

**Figure 1:**
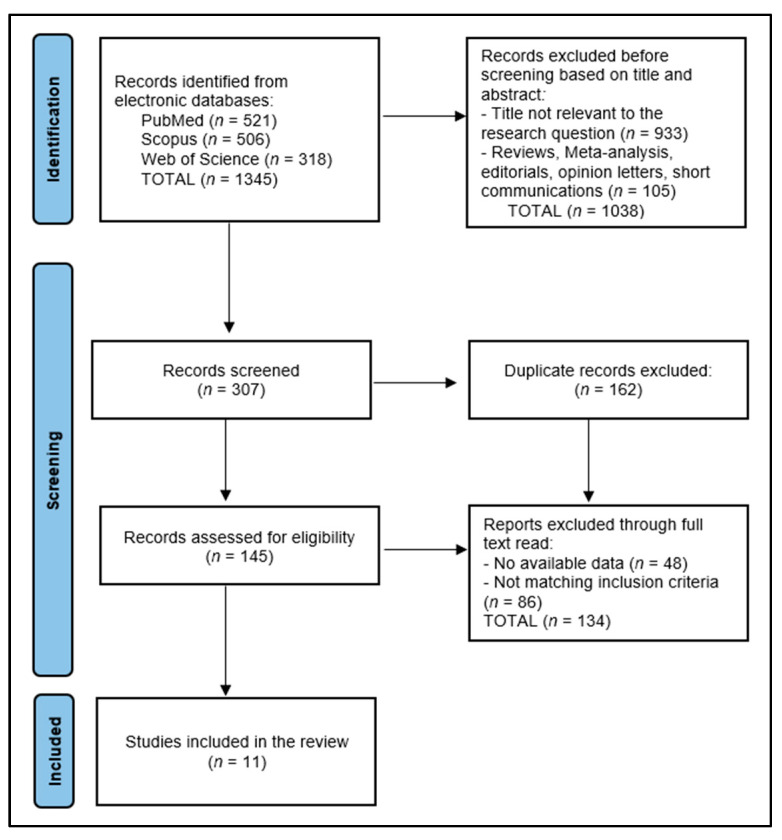
PRISMA flow diagram.

The data collection process for this systematic review commenced with the removal of 162 duplicate entries, followed by a rigorous screening of abstracts by two independent reviewers to assess each study’s relevance, based on predefined inclusion and exclusion criteria. Discrepancies between reviewers were resolved through discussion or, if necessary, consultation with a third reviewer to achieve consensus. The initial database search yielded a number of 1345 articles, from which 11 relevant studies were identified for inclusion in the final study, as presented in Figure 1.

### 2.5. Risk of Bias and Quality Assessment

For the systematic assessment of study quality and determination of risk of bias within the included studies, our review employed a dual approach, integrating both qualitative and quantitative evaluation methods. Initially, the quality of observational studies was evaluated using the Newcastle–Ottawa Scale [30], a widely recognized tool that assesses three critical dimensions: the selection of study groups, the comparability of these groups, and the ascertainment of either the exposure or outcome of interest for case–control or cohort studies, respectively. Each study is awarded stars in these categories, cumulating in a score that classifies the study quality as either low, medium, or high. This star system facilitates a nuanced evaluation of study quality, enabling the systematic identification of research that meets high methodological standards. To ensure the objectivity and reproducibility of our quality assessment process, each study was independently evaluated by two researchers. Discrepancies in quality assessment scores were resolved through discussion or, if necessary, consultation with a third researcher.

## 3. Results

### 3.1. Study Characteristics

The systematic review evaluated the impact of systemic treatments on outcomes and quality of life in patients with RAS-positive stage IV colorectal cancer, focusing on 11 distinct studies [31,32,33,34,35,36,37,38,39,40,41] ranging from 2011 to 2023. These studies were conducted across various countries, including France (Bennett et al. [31], Bertaut et al. [40]), the United States (Odom et al. [32]), multiple nations (Láng et al. [33], Yamaguchi et al. [35]), the United Kingdom (Seymour et al. [34]), Japan (Shitara et al. [36], Ooki et al. [39]), and Italy (Pietrantonio et al. [37], Raimondi et al. [38]), with Germany (Ballhausen et al. [41]) also contributing valuable data. Predominantly, these investigations were randomized trials, a design choice that underscores the rigorous methodology applied in assessing the efficacy and safety of systemic treatments within this patient population.

The quality of the studies, as delineated in Table 1, predominantly falls within the medium to high range, indicating a reasonable to excellent level of methodological rigor, data collection, and analysis. The high-quality ratings assigned to studies like those by Láng et al. [33] and Seymour et al. [34] in multinational and UK settings, respectively, along with Ooki et al. [39] in Japan, reflect the meticulous attention to detail and comprehensive data collection protocols these studies employed. Conversely, studies categorized with medium quality, although robust, suggest areas where methodological or reporting enhancements could further solidify their contributions to the literature.

This geographical and methodological diversity offers a broad spectrum of insights into the nuanced effects of systemic treatments on RAS-positive stage IV colorectal cancer patients. Notably, the studies conducted in Japan by Shitara et al. [36] and Ooki et al. [39] and the multinational studies by Láng et al. [33] and Yamaguchi et al. [35] provide a compelling narrative on the advancements in treatment strategies and their subsequent impact on patient outcomes and QoL across different healthcare systems.

### 3.2. Patients’ Characteristics

The systematic review analyzed studies employing varied systemic treatments in RAS-positive stage IV colorectal cancer. Bennett et al. [31] investigated panitumumab with FOLFOX4 (n = 284) and FOLFIRI (n = 263) against chemotherapy alone, demonstrating early integration of targeted therapy. Seymour et al. [34] compared irinotecan with panitumumab (IrPan, n = 230) to irinotecan alone, assessing EGFR pathway targeting benefits. Shitara et al. [36] explored sequencing by comparing regorafenib followed by cetuximab ± irinotecan (n = 51) against the reverse order (n = 50). Ballhausen et al. [41] examined panitumumab as maintenance therapy (n = 53) versus fluorouracil and folinic acid alone (n = 48), highlighting maintenance therapy’s role post-induction.

The patient populations across these studies were predominantly middle-aged to elderly, with reported mean or median ages ranging narrowly from 59 to 68 years across studies that disclosed age demographics (Bennett et al. [31], Odom et al. [32], Láng et al. [33], Seymour et al. [34], Yamaguchi et al. [35], Shitara et al. [36], Pietrantonio et al. [37], Raimondi et al. [38], Ooki et al. [39]).

Gender distribution within these studies highlighted a slight male predominance in the patient cohorts, with percentages of male participants ranging from 42% to 70% across studies that reported gender data (Bennett et al. [31], Odom et al. [32], Láng et al. [33], Seymour et al. [34], Yamaguchi et al. [35], Shitara et al. [36], Pietrantonio et al. [37], Raimondi et al. [38], Ooki et al. [39]).

Racial demographics were less frequently reported, with only a few studies providing data on the racial composition of their patient cohorts. Among those that did, a high percentage of white participants was noted, ranging from 90.5% to 100% in the studies that specified race (Bennett et al. [31], Odom et al. [32], Raimondi et al. [38], Ooki et al. [39]), as presented in Table 2.

### 3.3. Disease Characteristics

Disease characteristics across the reviewed studies uniformly underscored the advanced stage of colorectal cancer in patients with RAS mutations, demonstrating the studies’ focus on a subgroup with significant therapeutic challenges. RAS mutation specifics were consistently reported, with 100% KRAS wild-type in several studies, such as those by Bennett et al. [31], Láng et al. [33], and Seymour et al. [34]. For instance, Bennett et al. [31] and Seymour et al. [34] reported a high prevalence of colon involvement, with 60.8–65.8% and 27–36% of cases, respectively, further segmented into rectal cancer percentages, illustrating the broad anatomical distribution of primary tumors within this patient cohort. This diversity in tumor location underscores the heterogeneity of stage IV colorectal cancer and the necessity for tailored systemic treatment approaches.

The studies also detailed the extent of metastasis, a critical factor in stage IV disease, with liver metastases being particularly prevalent. For example, Bennett et al. [31] noted liver-only involvement in 17–21% of cases, while Seymour et al. [34] reported a higher range of liver involvement at 72–76%.

Performance status, predominantly rated using the Eastern Cooperative Oncology Group (ECOG) scale or the World Health Organization (WHO) performance status, was notably high across the studies, with a majority of patients maintaining a status of 0–1, indicating relatively preserved functionality despite advanced disease. For instance, Láng et al. [33] and Ooki et al. [39] reported ECOG 0–1 in over 90% of their cohorts, emphasizing the potential for aggressive systemic treatments in this population, as presented in Table 3.

### 3.4. Outcomes and Quality of Life

Table 4 presents a detailed compilation of baseline results, QoL follow-up results, complications/drop-out/survival, and study conclusions, emphasizing the quantitative aspects of the findings. For instance, Bennett et al. [31] reported baseline EQ-5D Health Status Index (HSI) mean scores ranging from 0.76 to 0.78 and EQ-5D Visual Analogue Scale (VAS) mean scores from 70.1 to 74.1, with a noted improvement in EQ-5D scores that was not deemed clinically meaningful. This study also highlighted a late dropout/completer rate varying between 29.8 and 70.2% across different treatment arms, concluding that the addition of panitumumab to chemotherapy did not significantly compromise QoL while notably improving disease-free survival (DFS).

Similarly, Odom et al. [32] presented Functional Assessment of Cancer Therapy-Colorectal (FACT-C) Score Mean ranges from 72.27 to 73.21 for the panitumumab plus Best Supportive Care (BSC) arm, and 71.84 to 71.91 for the BSC alone arm, with EQ-5D Index Mean scores between 0.68 and 0.73. The study observed improvements in FCSI and EQ-5D Index scores, particularly favoring the panitumumab + BSC in wild-type KRAS metastatic colorectal cancer, with early dropout rates of 38–42% for panitumumab + BSC and 68% for BSC alone. The study concluded that patients treated with panitumumab maintained better control of CRC symptoms and QoL compared with BSC alone.

Láng et al. [33] did not report baseline QoL scores, but noted worsened nausea and vomiting at week 16 and a worse change from baseline score for dyspnea in the FOLFIRI + Cetuximab arm. With a median survival of 25.7 months for cetuximab + FOLFIRI versus 16.4 months for FOLFIRI alone, the study concluded that adding cetuximab to FOLFIRI did not significantly impact GHS/QoL or social functioning, despite improved response rates and survival.

## 4. Discussion

### 4.1. Summary of Evidence

The systematic review of systemic treatments for patients with RAS-positive stage IV colorectal cancer reveals a complex landscape influenced by a multitude of factors, including patient demographics, tumor genetics, and the nuanced effects of different treatment modalities. A critical analysis of the data suggests that the efficacy of treatments such as panitumumab and cetuximab hinges significantly on the RAS mutation status of the tumor. Studies like Bennett et al. [31] and Odom et al. [32] underscore the tailored approach in treating mCRC, where targeted therapies in RAS wild-type patients do not compromise QoL significantly while improving survival outcomes. This specificity underscores the importance of genetic profiling in guiding treatment decisions, ensuring that patients receive the most effective and personalized therapy available.

However, the current findings also highlight the inherent challenges in managing stage IV colorectal cancer, particularly the balance between extending survival and maintaining quality of life. While treatments like cetuximab added to FOLFIRI have shown to not negatively impact QoL while improving PFS and OS in RAS wild-type mCRC patients, as observed in studies such as Yamaguchi et al. [35], the varied response rates and survival benefits across studies indicate a need for a deeper understanding of the interaction between tumor biology and treatment efficacy. The discrepancies in outcomes emphasize the complexity of treating advanced colorectal cancer and the need for ongoing research to identify which patients are most likely to benefit from specific systemic treatments.

The synthesis of findings across multiple studies underscores the significant impact of RAS mutations on the progression and treatment outcomes of stage IV colorectal cancer. Despite the challenges of managing such advanced disease, the high performance status maintained by a majority of patients (ECOG 0–1) supports the feasibility of aggressive systemic treatments. Key findings include a high prevalence of liver metastases and variable tumor locations, influencing treatment strategies and necessitating personalized approaches. Importantly, the studies highlight that while systemic treatments like cetuximab and panitumumab improve disease control without significantly compromising quality of life, the adverse effects related to these therapies, such as gastrointestinal and skin reactions, require careful management to optimize patient outcomes.

Nevertheless, complications and dropout rates reported across the studies further complicate the clinical decision-making process. The variability in patient adherence and tolerance to treatments, such as the differences in dropout rates between panitumumab plus BSC vs. BSC alone in the study by Odom et al. [32], raises questions about the real-world applicability of these therapies. These findings highlight the critical need for supportive care measures and patient education to enhance treatment adherence and manage side effects effectively, thereby potentially improving clinical outcomes.

The studies reviewed employed various quality of life measurement tools, including the EQ-5D, FACT-C, and QLQ-C30, each differing in focus and sensitivity. The EQ-5D is a generic instrument that may not capture colorectal cancer-specific issues as precisely as the FACT-C, which is tailored for colorectal cancer patients and includes specific subscales relevant to the condition. The QLQ-C30, while broadly applicable across cancers, also varies in the depth of cancer-specific impact it measures. These variations can affect comparability of QoL outcomes across studies due to differences in tool specificity, scoring interpretations, and cultural adaptations.

Moreover, the impact of systemic treatments on QoL remains a pivotal concern. While some studies report improvements or stable QoL scores post-treatment, the presence of significant adverse events, as noted in the comparison of complication rates between treatment arms, underscores the need for a balanced approach to treatment planning. This necessitates a careful evaluation of the potential benefits of treatment against the risk of adverse effects, emphasizing the importance of patient-centered care in the management of stage IV cancer, as it was proven as the most efficient method by recent studies [42,43].

The studies highlight that despite the high-performance status of patients (ECOG 0–1), indicating relatively preserved functionality, the prevalence of liver and other metastases demands aggressive treatment strategies. Notably, the uniform reporting of KRAS wild-type status in several studies underscores a specific patient subgroup with particular clinical characteristics. This data provides a critical foundation for future research aimed at optimizing treatment protocols and improving patient outcomes in this high-risk patient population.

The CodeBreaK 300 study, a phase 3 trial, demonstrated that sotorasib, in combination with panitumumab, significantly improves progression-free survival in patients with chemorefractory KRAS G12C-mutated metastatic CRC [44]. Key patient-reported outcomes revealed that both doses of sotorasib (960 mg and 240 mg) led to favorable changes from baseline in fatigue, pain, physical functioning, and global health status/quality of life compared to the control group. Specifically, the study highlighted a statistically significant improvement in pain and physical functioning scores at week 8 for both sotorasib groups, with the 960 mg dose also showing a significant improvement in global health status/quality of life. Additionally, time to deterioration analyses indicated a trend towards delayed deterioration in patients treated with sotorasib, particularly in the 240 mg dose group with respect to fatigue and physical functioning, underscoring the added benefit of sotorasib plus panitumumab in enhancing quality of life for patients with chemorefractory mCRC.

The study by Jiang et al. [45] provided a critical analysis comparing the efficacy of systemic treatments on QoL for patients with metastatic CRC, revealing that immunotherapy and targeted therapy significantly improved QoL over chemotherapy, with mean differences of 9.27 (95% CI: 3.96 to 14.6) and 4.04 (95% CI: 0.11 to 7.94), respectively. This contrasts with the CodeBreaK 300 study, which found both doses of sotorasib combined with panitumumab to offer benefits in QoL, with a notable improvement in global health status/quality of life (GHS/QoL) for the soto960 + pmab group by 9.17 points (95% CI: 2.10, 16.25). Jiang et al. emphasized the superiority of monotherapy, particularly cetuximab, in short-term QoL improvements over combination therapy and no active treatment, underscoring a paradigm shift towards personalized medicine in mCRC treatment, where individual gene expression profiles might dictate the choice of therapy for optimizing both survival and quality of life.

The review by Battaglin et al. [46] highlighted the efficacy of panitumumab, either in combination with chemotherapy as a first- or second-line treatment or as a monotherapy in advanced lines for chemorefractory patients with RAS wild-type metastatic CRC, according to the analyzed trials [47,48,49]; however, quality of life was not directly assessed in these trials. Among other conclusions Battaglin et al., underscored the manageable toxicity profile of panitumumab and its favorable impact on the QoL for patients, including those who are frail and elderly. The study detailed emerging treatment scenarios leveraging panitumumab, such as intensified chemotherapy regimens aimed at converting initially unresectable mCRC to resectable states and employing maintenance treatment strategies. Furthermore, Battaglin et al. discussed the ongoing efforts to understand the mechanisms behind both primary and acquired resistance to panitumumab, emphasizing the need for a more comprehensive molecular characterization of RAS WT tumors. This includes assessing additional mutational and clinico-pathological features, like BRAF status [50] and tumor sidedness, and developing novel technologies to capture the dynamic genomic heterogeneity of mCRC under targeted treatment. The advancements in this field, as noted by the authors, necessitate a prospective validation of new predictive biomarkers in RAS WT mCRC to refine patient selection further and develop novel molecularly tailored treatment strategies to optimize outcomes and benefit patients.

The study conducted by Murakawa et al. [51] embarked on a retrospective evaluation of 90 patients with inoperable colon cancer undergoing chemotherapy, revealing that patients with inoperable colon cancer at first onset exhibited worse OS compared to those with inoperable colon cancer at the time of relapse. However, the study identified no correlation between OS and length of hospitalization for both patient groups, whereas a strong correlation was observed between OS and both outpatient consultation times and hospital-free survival. Intriguingly, length of hospitalization and hospital-free survival represented about 8% and 90% of their OS, respectively, underscoring their significance as objective measures for assessing QoL in this patient population.

It is also important to emphasize that dermatologic toxicity, particularly related to treatments like cetuximab, significantly impacts the quality of life in patients with RAS-positive metastatic colorectal cancer. Our review finds that while systemic therapies provide substantial disease control, they also introduce challenges such as skin reactions, which are not as thoroughly assessed as hematologic or biochemical toxicities. For example, Láng et al. noted early skin reactions in patients receiving cetuximab, which, although not significant, affect global health status scores. Chiang et al. [52] underscore the prevalence of severe skin reactions in 50–70% of patients using anti-EGFR antagonists. Chiang et al. also highlight that while skin toxicity occurs frequently with these treatments, it did not predict the effectiveness of the anti-EGFR medication in their study, pointing to a complex relationship between side effects and therapeutic outcomes. Implementing standardized dermatologic assessment tools in clinical trials will help in quantifying these impacts more accurately, leading to improved management strategies and ultimately enhancing patient care and adherence to therapies. Therefore, the critical analysis of systemic treatments for RAS-positive stage IV colorectal cancer underscores the complex interplay between extending survival and preserving quality of life. The review highlights the importance of personalized treatment strategies based on tumor genetics, patient preferences, and the careful management of treatment-related complications. It advocates for a multidisciplinary approach to treatment planning, incorporating genetic profiling, patient education, and supportive care to optimize outcomes for patients with advanced colorectal cancer.

### 4.2. Limitations

This systematic review faced limitations, including a language restriction to English, potentially overlooking pertinent studies in other languages or published subsequently. The exclusion of unpublished trial studies and grey literature could introduce publication bias, skewing results towards studies with positive outcomes. Furthermore, the reliance on published data for analysis might not fully capture adverse effects or long-term outcomes, as these can be underreported. The heterogeneity among studies regarding patient populations, treatment regimens, location of tumors, and outcome measures complicates the direct comparison of results, affecting the generalizability of the findings. Additionally, the qualitative assessment of study quality, despite using established tools, remains subjective and may introduce bias in evaluating the methodological rigor of the included studies.

## 5. Conclusions

In conclusion, the review highlights the efficacy of various systemic therapies, particularly the combination of targeted therapies like panitumumab and cetuximab with chemotherapy regimens such as FOLFOX4 and FOLFIRI. The findings demonstrate that these combinations do not significantly affect QoL negatively, while offering substantial benefits in disease-free survival. Furthermore, the study emphasizes the importance of personalized treatment plans and the need for further research aimed at improving survival and QoL outcomes. Overall, the review strongly supports the integration of targeted therapies with chemotherapy in the treatment paradigm for RAS-positive stage IV colorectal cancer, advocating for personalized approaches to enhance patient outcomes in this challenging disease setting.

## Figures and Tables

**Table 1:** Study characteristics.

Study & Author	Country	Study Year	Study Design	Study Quality
1 Bennett et al. [31]	France	2011	Randomized trial	Medium
2 Odom et al. [32]	United States	2011	Randomized trial	Medium
3 Láng et al. [33]	Multinational	2013	Randomized trial	High
4 Seymour et al. [34]	United Kingdom	2013	Randomized trial	High
5 Yamaguchi et al. [35]	Multinational	2017	Randomized trial	Medium
6 Shitara et al. [36]	Japan	2019	Randomized trial	High
7 Pietrantonio et al. [37]	Italy	2020	Randomized trial	Medium
8 Raimondi et al. [38]	Italy	2020	Randomized trial	Medium
9 Ooki et al. [39]	Japan	2022	Randomized trial	High
10 Bertaut et al. [40]	France	2022	Randomized trial	Medium
11 Ballhausen et al. [41]	Germany	2023	Randomized trial	Medium

**Table 2:** Patient characteristics.

Study Number	Study Arm (s)	Control Arm (s)	Age, Years (Mean/Median)	Gender Distribution (Male)	Race (White)
1 Bennett et al. [31]	Panitumumab + FOLFOX4 (n = 284) Panitumumab + FOLFIRI (n = 263)	FOLFOX4 alone (n = 292) FOLFIRI alone (n = 267)	60.1–60.6	66.5% (Panitumumab + FOLFOX4) 63.7% (FOLFOX4 alone) 60.5% (Panitumumab + FOLFIRI) 64.0% (FOLFIRI alone)	90.5–97.0%
2 Odom et al. [32]	Panitumumab plus BSC (n = 188)	BSC alone (n = 175)	61–62	65%	98–99%
3 Láng et al. [33]	FOLFIRI + Cetuximab (n = 300)	FOLFIRI (n = 327)	60	62–63%	NR
4 Seymour et al. [34]	IrPan (n = 230)	Irinotecan (n = 230)	63–64	69–70%	NR
5 Yamaguchi et al. [35]	Cetuximab + FOLFIRI (n = 170)	FOLFIRI alone (n = 181)	59–60	63.5–65.7%	NR
6 Shitara et al. [36]	Regorafenib followed by Cetuximab ± Irinotecan (R-C arm) (n = 51)	Cetuximab ± Irinotecan followed by Regorafenib (C-R arm) (n = 50)	65–68	61–66%	NR
7 Pietrantonio et al. [37]	Capecitabine plus temozolomide (CAPTEM) (n = 43)	FOLFIRI (n = 43)	67–70	CAPTEM: 42% FOLFIRI: 56%	100%
8 Raimondi et al. [38]	Panitumumab plus 5-FU/LV (Arm A) (n = 107)	Panitumumab (Arm B) (n = 10.3)	63.5	65.7%	100%
9 Ooki et al. [39]	Cetuximab plus chemotherapy (n = 128)	NR	66	68.0%	100%
10 Bertaut et al. [40]	Bevacizumab + chemotherapy (mFOLFOX6 or FOLFIRI) (n = 65)	Cetuximab + chemotherapy (mFOLFOX6 or FOLFIRI) (n = 67)	NR	NR	NR
11 Ballhausen et al. [41]	Panitumumab (n = 53)	Fluorouracil + Folinic Acid (n = 48)	NR	NR	NR

NR—Not Reported; FOLFOX4—Folinic Acid, Fluorouracil, Oxaliplatin; FOLFIRI—Folinic Acid, Fluorouracil, Irinotecan; BSC—Best Supportive Care; IrPan—Irinotecan, Panitumumab; CAPTEM—Capecitabine, Temozolomide; 5-FU/LV—5-Fluorouracil and Leucovorin.

**Table 3:** Disease characteristics.

Study Number	Disease Duration/Follow-Up	Performance Status	Primary Tumor	Metastasis Site (s)	RAS Mutation
1 Bennett et al. [31]	Follow-up monthly until disease progression	ECOG 0–1: 94.5–96.2%	Colon: 60.8–65.8% Rectum: 35.6–39.2%	Liver only: 17–21% Liver + other: 63–68% Other only: 13–15%	Wild-type KRAS: 100%
2 Odom et al. [32]	Time since primary diagnosis: 31–33 months (mean) Time since metastatic disease: 19–24 months (mean)	ECOG 0: 35–48% ECOG 1: 40–52% ECOG 2: 11–15%	Colon: 67% Rectum: 33%	NR	Wild-type: 60.6% Mutant: 39.4%
3 Láng et al. [33]	Follow-up every 3 months until disease progression	ECOG 0: 57–59% ECOG 1: 37–39% ECOG 2: 4%	Colon: 67–70% Rectum: 30–33%	Liver metastases only: 21–23%	Wild-type KRAS: 100%
4 Seymour et al. [34]	Median follow-up 25.4 months	WHO 0–1: 94% WHO 2: 6%	Right colon: 27–32% Left colon: 29–36% Rectum: 35–36%	Liver: 72–76% Lung: 50–54%	Wild-type KRAS: 100%
5 Yamaguchi et al. [35]	Follow-up every 8 weeks, up to 32–40 weeks	ECOG 0: 55.9–60.2% ECOG 1: 35.9–41.2% ECOG 2: 2.9–3.9%	NR	Liver metastases: 23.8–24.7% More than two sites involved: 8.2–13.3%	Wild-type KRAS: 100%
6 Shitara et al. [36]	Median follow-up 29 months. Assessment every 4 weeks.	ECOG PS 0: 67% in R-C, 78% in C-R ECOG PS 1: 33% in R-C, 22% in C-R	Left-side: 75% in R-C, 86% in C-R; Right-side: 25% in R-C, 14% in C-R	Liver: 63% in R-C, 62% in C-R; Lung: 59% in R-C, 46% in C-R; Lymph node: 41% in R-C, 48% in C-R; Peritoneum: 22% in R-C, 18% in C-R	Wild-type in circulating tumor DNA (ctDNA) at study entry: 90% in R-C arm, 88% in C-R arm; Mutant: 8% in both arms
7 Pietrantonio et al. [37]	Median follow-up 30.5 months. QoL assessment every 8 weeks.	ECOG 0: 56% in CAPTEM, 51% in FOLFIRI; ECOG 1: 44% in CAPTEM, 49% in FOLFIRI	Right side: 35% in CAPTEM, 40% in FOLFIRI; Left side: 65% in CAPTEM, 60% in FOLFIRI	Synchronous metastases 72% (CAPTEM) vs. 67% (FOLFIRI) >1 metastatic sites 58% (CAPTEM) vs. 65% (FOLFIRI)	RAS-mutated, MGMT-methylated mCRC 100%
8 Raimondi et al. [38]	Follow-up for 40 weeks. Assessments every 8 weeks.	ECOG 0: 73.8% ECOG 1: 26.2%	Left-sided (84.3%) vs. Right-sided (15.7%).	Liver-limited disease in 35.2% of patients, peritoneal metastasis in 22.9%.	Wild-type RAS: 100%
9 Ooki et al. [39]	Assessments at 2, 4, 8, 16, and 24 weeks for QoL and every 8 weeks for radiologic assessments.	ECOG 0: 83.6% ECOG 1: 16.4%	Colon: 64.8% Rectum: 35.2%	≥2 metastases 64.0%	Wild-type KRAS: 100%
10 Bertaut et al. [40]	Assessment 1 and 3 (approximately 6 and 18 weeks after initial treatment) Median follow-up time 4.1 months for bevacizumab group vs. 1.7 months cetuximab group	WHO 0–1: 100%	NR	NR	Wild-type RAS: 100%
11 Ballhausen et al. [41]	10 treatment cycles	ECOG 0–1: 100%	NR	NR	Wild-type RAS: 100%

NR—Not Reported; ECOG—Eastern Cooperative Oncology Group; QoL—Quality of Life; FOLFOLFIRI—Folinic Acid, Fluorouracil, Irinotecan; CAPTEM—Capecitabine, Temozolomide; WHO—World Health Organization; MGMT—methyl-guanine-DNA-methyltransferase.

**Table 4:** Outcomes and quality of life.

Study Number	Baseline Results	QoL Follow-Up Results	Complications/Drop-out/Survival	Conclusions
1 Bennett et al. [31]	EQ-5D HSI Mean: 0.76–0.78 EQ-5D VAS Mean: 70.1–74.1	Improvement in EQ-5D scores not clinically meaningful	Late dropout/completer: 48–52% (Panitumumab + FOLFOX4); 37.4–62.6% (FOLFOX4 alone); 39.7–60.3% (Panitumumab + FOLFIRI); 29.8–70.2% (FOLFIRI alone)	Addition of panitumumab to chemotherapy (FOLFOX4 or FOLFIRI) in wild-type KRAS mCRC did not compromise QoL significantly and improved significantly the DFS.
2 Odom et al. [32]	FCSI Score Mean: 72.27–73.21 (Panitumumab + BSC) 71.84–71.91 (BSC alone) EQ-5D Index Mean: 0.68–0.73	Improvement in FCSI and EQ-5D Index scores favored Panitumumab + BSC, especially in wild-type KRAS mCRC	Early dropout: 38–42% (Panitumumab + BSC), 68% (BSC alone) Late dropout/completer: 57–62% (Panitumumab + BSC), 31–32% (BSC alone)	Panitumumab-treated patients with wild-type KRAS mCRC maintained better control of CRC symptoms and quality of life compared with BSC alone.
3 Láng et al. [33]	NR	Worsened nausea and vomiting at week 16 (FOLFIRI vs. FOLFIRI + Cetuximab: 14.25 vs. 9.08). A worse change from baseline score for dyspnea in the FOLFIRI + Cetuximab arm. Early skin reactions in patients receiving cetuximab did not significantly affect these QoL scales	Median survival 25.7 (cetuximab + FOLFIRI) vs. 16.4 months (FOLFIRI) (HR 1.68). Constant and similar compliance rates. Better tumor response 58% (cetuximab + FOLFIRI) vs. 40% FOLFIRI	Adding cetuximab to FOLFIRI did not significantly impact GHS/QoL or social functioning, despite improved response rates and survival.
4 Seymour et al. [34]	NR	Grade 3 or worse diarrhea (29% vs. 18%), skin toxicity (19% vs. 0%), lethargy (21% vs. 11%), infection (19% vs. 10%), and hematological toxicity (22% vs. 12%) more common in IrPan group.	Progression-free survival longer in IrPan group (HR 0.78). Higher response rate in IrPan group (34% vs. 12%). Five treatment-related deaths reported (1.0%).	Adding panitumumab to irinotecan did not improve overall survival for patients with wild-type KRAS tumors, despite longer progression-free survival and higher response rate.
5 Yamaguchi et al. [35]	Cetuximab + FOLFIRI vs. FOLFIRI GHS/QoL 60.9 vs. 61.9 Physical functioning 77.4 vs. 78.7 Fatigue 32.6 vs. 33.2 Nausea and vomiting 7.7 vs. 6.5 Pain 23.3 vs. 23.6	Cetuximab + FOLFIRI vs. FOLFIRI GHS/QoL 65.0 vs. 68.6 Physical functioning 80.2 vs. 80.0 Fatigue 29.3 vs. 31.6 Nausea and vomiting 8.7 vs. 12.0 Pain 14.6 vs. 14.7	High drop-out rate.	Adding cetuximab to FOLFIRI did not negatively impact QoL, while improving PFS, OS, and ORR in patients with RAS wild-type mCRC. Changes in GHS/QoL and social functioning from baseline to week 8 were similar irrespective of whether patients experienced early skin reactions.
6 Shitara et al. [36]	NR	Average EQ-5D between the two arms after treatment (difference = −0.011).	Median OS significantly longer in R-C (17.4 months) vs. C-R (11.6 months), HR for OS was 0.61 (95% CI, 0.39–0.96). No unexpected safety signals were observed.	Regorafenib treatment resulted in worse mobility, pain/discomfort, self-care, and usual activity scores compared to cetuximab after 4 weeks in both treatment periods, particularly in the C-R arm.
7 Pietrantonio et al. [37]	NR	QLQ-30 showed a significantly better QoL in CAPTEM arm +5.42 vs. −17.19 at 8 weeks, and +3.57 vs. −11.67 at 16 weeks. FACT-C showed a significantly better change from baseline in the CAPTEM arm +0.19 vs. −7.06 at 8 weeks, and −2.07 vs. −9.74 at 24 weeks.	Median PFS: 3.5 months for both arms; Median OS: 9.5 months for CAPTEM and 10.6 months for FOLFIRI; Grade 3 treatment-related adverse events higher in FOLFIRI (47.6%) vs. CAPTEM (16.3%) Mortality 65.1% in CAPTEM arm vs. 72.1% in FOLFIRI	CAPTEM regimen failed to show superiority over FOLFIRI. Better QoL in the CAPTEM arm.
8 Raimondi et al. [38]	NR	Global QoL worsened in 40.9% in panitumumab plus5-FU/LV arm vs. 29.5% in panitumumab arm.	Compliance at baseline was high in both arms (Arm A: 91.5%, Arm B: 92.0%). Rates of patients completing questionnaires decreased over time.	Induction with oxaliplatin-containing chemotherapy plus anti-EGFRs leads to transient QoL deterioration, with overall recovery during maintenance, highlighting the impact of treatment deintensification on health-related QoL.
9 Ooki et al. [39]	GHS/QoL 69	ETS associated with improved QoL scores between baseline and 8 weeks: +5.86 for GHS/QoL, +26.73 for physical functioning, and +13.58 for social functioning in symptomatic patients.	Early tumor shrinkage achieved in 64.1% treated with cetuximab + chemotherapy. PFS was 10.8 months. 2-year OS 42.5% in symptomatic patients vs. 77.8% asymptomatic	ETS in patients treated with first-line cetuximab plus chemotherapy is associated with significant improvements in QoL for symptomatic patients, underscoring the importance of ETS in treatment efficacy and patient well-being.
10 Bertaut et al. [40]	GHS/QoL 66.7	GHS/QoL 62.5 Bevacizumab vs. 50.0 Cetuximab.	Median time to deterioration was 6 months. Diarrhea QLQ-C30 score is significantly higher in Bevacizumab.	No relevant impairment of patients QoL between the 2 treatment arms.
11 Ballhausen et al. [41]	GHS/QoL 59.8	GHS/QoL 59.8	Appetite loss and diarrhea worsened in the Pman group.	The addition of Pmab to FU/FA as maintenance therapy prolongs DFS without negative impact on QoL.

NR—Not Reported; EQ-5D HSI—EuroQol 5-Dimension Health State Index; FCSI—Functional Assessment of Chronic Illness Therapy Score; GHS/QoL—Global Health Status/Quality of LifeQLQ-30—Quality of Life Questionnaire C-30FACT-C—Functional Assessment of Cancer Therapy-Colorectal; ETS—Early Tumor Shrinkage; DFS—Disease-Free Survival; PFS—Progression-Free Survival; OS—Overall Survival; ORR—Objective Response Rate; HR—Hazard Ratio; CI—Confidence Intervalm; CRC—Metastatic Colorectal Cancer; KRAS—Kirsten Rat Sarcoma Viral Oncogene Homolog5-FU/LV—5-Fluorouracil and LeucovorinQLQ-C30—Quality of Life Questionnaire-Core 30Pmab—Panitumumab; FU/FA—Fluorouracil and Folinic Acid.

## Data Availability

Not applicable.
